# Circulating levels of cytokines, chemokines and growth factors in patients with achalasia

**DOI:** 10.3892/br.2021.1468

**Published:** 2021-09-10

**Authors:** Anna Panza, Andrea Fontana, Orazio Palmieri, Antonio Merla, Massimiliano Copetti, Antonello Cuttitta, Giuseppe Biscaglia, Annamaria Gentile, Angelo Andriulli, Anna Latiano

**Affiliations:** 1 Fondazione IRCCS Casa Sollievo della Sofferenza, Gastroenterology Unit, I-71013 San Giovanni Rotondo, Foggia, Italy; 2 Fondazione IRCCS Casa Sollievo della Sofferenza, Unit of Biostatistics, I-71013 San Giovanni Rotondo, Foggia, Italy; 3 Fondazione IRCCS Casa Sollievo della Sofferenza, Unit of Thoracic Surgery, I-71013 San Giovanni Rotondo, Foggia, Italy

**Keywords:** achalasia, inflammation, cytokines, chemokines, growth factor

## Abstract

Idiopathic achalasia is a disease that is characterized by the absence of peristalsis and incomplete relaxation of the lower esophageal sphincter, which is accompanied by dysphagia, regurgitation, chest pain and weight loss. The role of inflammatory infiltrates in the pathogenesis of achalasia remains controversial, although the infiltrating cell profile in the tissue has been previously characterized histologically and immunohistochemically. The present study aimed to evaluate the serum levels of 27 protein biomarkers to determine their association with achalasia and the clinical disease characteristics. The cytokine, chemokine and growth factor serum profiles of 68 patients with achalasia and 39 healthy individuals were explored using the 27-Bio-Plex Pro Human Cytokine assay. Reductions in the levels of inflammatory mediators IL-1β, IL-2, IL-5, IL-6, IL-8, IL-10, IL-12p70, IL-13, IL-15, IL-17, fibroblast growth factor, granulocyte colony-stimulating factor, granulocyte-macrophage colony-stimulating factor, interferon-γ, monocyte chemoattractant protein-1, macrophage inflammatory protein-1 (MIP-1)α and MIP-1β, regulated upon activation normal T cell expressed and presumably secreted, TNF-α and VEGF were detected in the serum samples of patients with achalasia compared with those in the control group (P<0.05). However, significant associations between the expression in the levels of inflammatory factors and clinical characteristics of the patients were not found (P>0.05). These results suggest that achalasia is a disease that has a local but not a systemic inflammatory pattern. Further studies are required to improve the current understanding of the mechanism underlying this disease.

## Introduction

Idiopathic achalasia is a motor disease of the esophagus characterized by the absence of peristalsis and incomplete relaxation of the lower esophageal sphincter. The most common clinical symptoms of achalasia include dysphagia, regurgitation, chest pain and weight loss. Currently available therapeutic options include medical treatment (such as calcium channel blockers and nitrates), botulinum toxin injection, pneumatic dilation, Heller myotomy and peroral endoscopic myotomy (POEM) (1). In particular, POEM is emerging as the treatment of choice for a significant proportion of patients (1). The etiology and pathophysiology of achalasia remain poorly understood, although it has been associated with a variety of putative mechanisms, including infection, autoimmune reactions, inflammatory processes and genetic factors (2,3). In addition, a possible association between achalasia and infection has been suggested. Several types of causal viruses, including herpes simplex virus, bornavirus, varicella zoster, measles and human papilloma virus, have all been documented to be involved (4). The contribution of human leukocyte antigen class II genes to the susceptibility of achalasia has also been demonstrated previously (3,5). Achalasia has been associated with the functional loss of myenteric plexus ganglion cells and neurons in the distal esophagus and with substantial immune cell infiltration that consists predominantly of T lymphocytes, which appears to decrease alongside disease progression (6). Moreover, the detection of circulating anti-myenteric plexus autoantibodies in patients with achalasia and its occurrence in association with other autoimmune diseases (7) supports the role of an inflammatory component mediated by the immune system within this disorder. Characterization of infiltrating cells in the tissues (muscle specimens) has largely been performed using histological or immunohistochemical analyses, which produced conflicting reports (7,8). Conversely, studies investigating the involvement of circulating levels of inflammatory mediators, cytokines, chemokines and growth factors in patients with achalasia remain insufficient (9,10).

Therefore, the present study aimed to characterize the systemic inflammatory patterns in the serum of patients with achalasia and healthy individuals. The aim of the present study was to explore the role of cytokines, chemokines and growth factors in the pathogenesis of achalasia, to gain some insight into the disease mechanisms and etiology of the disease.

## Patients and methods

### 

#### Patient enrolment and serum collection

A total of 68 patients (35 male and 33 female; age range, 27-84 years; median age, 59 years) diagnosed clinically with achalasia and confirmed by manometry were enrolled between October 2014 and March 2018 at the Division of Gastroenterology of ‘Casa Sollievo della Sofferenza’ Hospital (San Giovanni Rotondo, Italy). The study protocol followed the ethical guidelines of the Declaration of Helsinki (11), was approved by the Institutional Ethics Committee of the ‘Casa Sollievo della Sofferenza’ hospital (approval no. 139/CE on 28/10/2014) and all patients provided written informed consent. Esophageal manometry was performed using a low compliance perfusion system (12), where the manometric criteria used for achalasia diagnosis included esophageal aperistalsis and poor lower esophageal sphincter relaxation (13).

The medical records were also reviewed using a validated questionnaire. Clinical data regarding symptoms of regurgitation, dysphagia, chest pain and weight loss were collected and classified by following the scoring system previously described by Eckardt *et al* (14)*.* Concomitant autoimmune diagnosis, including uveitis, type I diabetes, rheumatoid arthritis and thyroid diseases, pneumatic dilatation of the esophagus and/or surgical therapy were reported. The healthy control group was comprised of 39 age- and sex-matched healthy blood donors (20 male and 19 female; age range, 30-79 years; median age, 59 years), who were recruited in the same time period and volunteered to donate to the blood bank of ‘Casa Sollievo della Sofferenza’ Hospital. The exclusion criteria for these samples were diagnoses of comorbidities, such as diabetes, autoimmune disease or any associated inflammatory or infectious diseases and administration of treatment with anti-inflammatory drugs. Written informed consent was obtained from all subjects. Blood samples from the patients and controls were collected from the Laboratory of Research, Division of Gastroenterology of ‘Casa Sollievo della Sofferenza’ Hospital and placed into Vacutainer^®^ Plus Plastic SST™ tubes with Gel for Serum Separation (BD Biosciences). The samples were then centrifuged at 1,620 x g for 15 min at room temperature before the sera were aliquoted into NUNC-cryovial tubes (Thermo Fisher Scientific Inc.) and stored at -80˚C until the time of analysis to prevent protein degradation. None of the serum samples had been previously thawed prior to thawing for the assay.

#### Cytokines, chemokines and growth factor measurement

A panel of 27 cytokines and chemokines were measured in the serum samples in duplicate using the Bio-Plex Pro Human Cytokine assay in 96-well plates (Bio-Rad Laboratories, Inc.), according to the protocols of the manufacturer. The Bio-Plex Pro Human Cytokine assay panel integrates a network of biologically relevant cytokines and chemokines in a single assay. It applies a standard sandwich ELISA method, which enables the simultaneous quantification and analysis of the following 27 cytokines, chemokines and growth factors: IL-1β, IL-1 receptor antagonist, IL-2, IL-4, IL-5, IL-6, IL-7, IL-8, IL-9, IL-10, IL-12p70, IL-13, IL-15, IL-17, Hu eotaxin, basic fibroblast growth factor (bFGF), granulocyte colony-stimulating factor (GCSF), granulocyte-macrophage colony-stimulating factor (GM-CSF), interferon (IFN)-γ, IFN-γ-induced protein 10 (IP-10), monocyte chemotactic protein (MCP)-1, macrophage inflammatory protein (MIP)-1α, platelet-derived growth factor (PDGF), MIP-1β, regulated upon activation normal T cell expressed and presumably secreted (RANTES), TNF-α and vascular endothelial growth factor (VEGF).

The appropriate analyte standards and samples were diluted in standard diluent and sample diluent, respectively. The median coefficient of variation was set at <25% for all cytokines analyzed. Bio-Plex Manager version 6.1 (Bio-Rad Laboratories, Inc.) was used for data analyses. Protein concentrations were calculated using the appropriate standard curves.

#### Statistical analysis

Clinical characteristics of patients with achalasia and healthy individuals were presented as the mean ± standard deviation, and as observed frequencies (and percentages) for continuous and categorical variables. Distribution of protein concentration in sera was evaluated for each considered analyte using quantile-quantile plots and the distribution of data was assessed using a Shapiro-Wilk test. Due to log-normal distributions, comparisons of mean protein concentrations between the patient and control groups were performed using an unpaired two-samples t-test of the log-transformed values (log-pg/ml). P-values were also adjusted for multiple comparisons using Bonferroni correction. Two-sided P<0.05 was considered to indicate a statistically significant difference. For the concentration of those cytokines, chemokines and growth factors that could be detected but not necessarily quantified as an exact value, a visual evaluation approach proposed by the Expert Working Group of the International Conference on Harmonisation of Technical Requirements for Registration of Pharmaceuticals for Human Use was performed (15). This is because the Bio-Plex Pro Human Cytokine assay has limits of quantification, which is defined as the lowest and the highest amount of analyte in a sample that can be quantitatively determined with suitable precision and accuracy. These unquantified concentrations were replaced by suitable values, which were estimated using the following protocol: i) For each analyte, the quantification limit was determined by observing the minimum and maximum concentration values observed in the data sample; ii) in cases where the unquantified concentration was below the limit of quantification, this was replaced by one that is 10% lower than the minimum detected value; and iii) otherwise, this would be replaced by one that is 10% higher than the maximum detected value. All statistical analyses were performed using R Core Team (16) with the following packages: Tableone (github.com/kaz-yos/tableone), tidyr (github.com/tidyverse/tidyr), ggplot2 (github.com/tidyverse/ggplot2).

## Results

### 

#### Clinical characteristics of the patients

The general and clinical characteristics of patients with achalasia are summarized in Table I. In total, 35 patients (51.5%) underwent surgical myotomy and 18 patients (26.5%) were successfully managed only by pneumatic dilatation. In addition, 15 patients (22%) underwent both esophagomyotomy and pneumatic dilatation, whilst 21 patients (30.9%) developed megaesophagus.

#### Comparison of cytokines, chemokines and growth factor concentrations in the sera of patients with achalasia and healthy individuals

**Figure 1:**
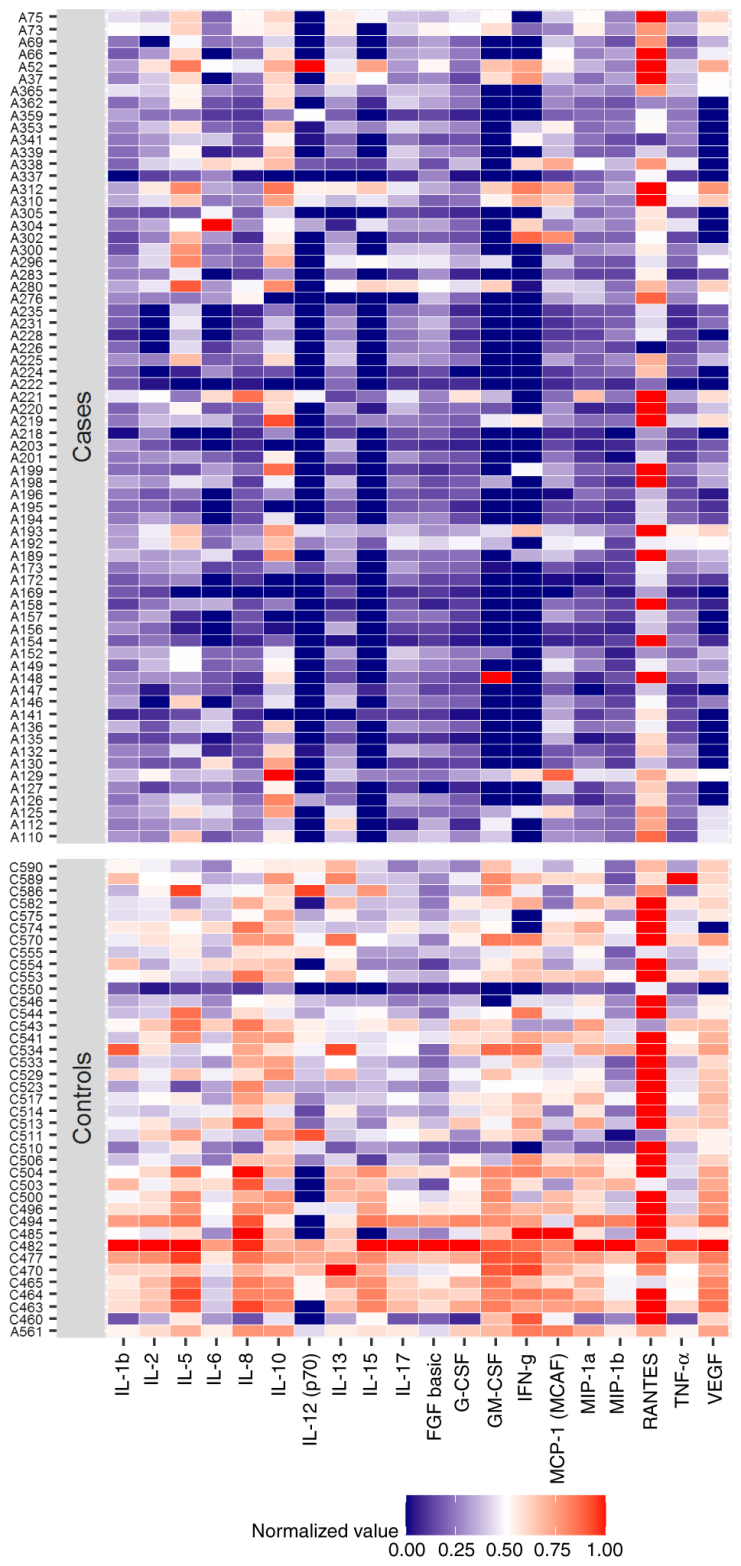
Heatmap of normalized protein concentrations in the sera of patients with achalasia and the controls.

Analysis of the concentrations of markers between the two study groups detected reduced levels of several cytokines, namely IL-1β, IL-2, IL-5, IL-6, IL-8, IL-10, IL-12p70, IL-13, IL-15, IL-17, bFGF, G-CSF, GM-CSF, IFN-γ, MCP-1, MIP-1α, MIP-1β, RANTES, TNF-α and VEGF, in the patients with achalasia compared with those in the control group (all P<0.001; Table II). Additionally, limits of quantification were detected for IL-6, IL-12p70, IL-15, GM-CSF, IFN-γ, VEGF and RANTES in >15% of the observations (Table SI). A heatmap of the normalized protein concentrations (that is the values from 0 to 1), which resulted in significantly different between patients and controls, is depicted in Fig. 1. In addition, to evaluate if the inflammatory profile differed between the cohorts, the 27 markers were re-grouped into the ‘chemokines’, ‘anti-inflammatory’, ‘pro-inflammatory’ and ‘growth factors’ categories. However, no significant changes were observed (data not shown).

#### Association between serum levels of cytokines, chemokines and growth factors and clinical data in achalasia

To assess the relationship between the tested serum levels and the clinical parameters of the patients (sex, dysphagia, esophageal regurgitation, chest pain, weight loss, autoimmune conditions, pneumatic dilatation, surgical myotomy and megaesophagus), an association analysis was performed. After the classification of the patients with achalasia were classified according to sex, the serum levels of bFGF were found to be higher in male patients compared with those in female patients (P=0.001). Significant changes in the evaluated proteins after re-categorization of the patients into the other remaining clinical characteristics were not found (P>0.05; data not shown).

## Discussion

A number of hypotheses point toward a multifactorial etiopathogenesis of achalasia (2,17,18).

One of those was suggested to be viral and autoimmune factors triggering inflammatory changes within the tissue with ensuing damage to the myenteric plexus (2). It has been hypothesized that various pathogens, including herpes simplex virus, varicella zoster, measles and human papilloma virus, may play a causative role in triggering tissue inflammation (4); however, none have been categorically proven to be a causal agent, since not all infected patients develop the disease (4). Chronic viral infection may trigger aberrant immune responses, where under appropriate genetic and environmental settings, loss of inhibitory neurons in the myenteric plexus occurs in the lower esophageal sphincter (LES), which releases vasoactive intestinal peptide and nitric oxide synthase (NOS), resulting in failure of LES muscle relaxation and loss of esophageal peristalsis (19). Although the pathophysiological factors responsible for alterations in enteric innervation remain unknown, the presence of lymphocytic infiltration of the myenteric plexus, occurrence of circulating antimyenteric neuronal antibodies and association with antigens of the class II major histocompatibility complex all support the existence of an immuno-inflammatory mechanism underlying neuronal cell loss. Involvement of cytokines and inflammatory mediators may be responsible for changes in the myenteric neuronal phenotype (6). Cytokines and chemokines released into the surrounding myenteric area can affect gene expression in the enteric neurons, such as NOS, consequently affecting LES function (20). In addition, they may serve an important role in nerve regeneration (21) and are able to activate microglia, leading to neuronal excitation or neuronal loss (22,23). The significant decrease in the number of myenteric neurons in the distal esophagus and the LES, in addition to the association of this reduction with inflammatory cell infiltration, has been previously described through histological examination of the resected specimens of patients with achalasia, and was hypothesized to represent a secondary phenomenon (24). At the early stages of the disease, the inflammatory infiltrates in the esophageal myenteric plexus are predominantly composed of the Th1, Th2 and regulatory cell subsets (6). In addition, Th17 and Th22 cells have also been reported to be the main cellular component in the LES muscle (25). Recently, a CD4^+^-dominant inflammatory T-cell population in the infiltrate of histological specimens at all stages of the disease and at all levels of the esophagus was found (8).

Few studies have measured the levels of circulating inflammatory biomarkers, namely cytokines, chemokines and growth factors, and deduced their differences between patients with achalasia and controls. Wang *et al* (9) previously measured the expression of IL-17 and IL-22 in the serum, which was collected before and during peroral endoscopic myotomy. It was found that the levels of IL-17 and IL-22 were significantly increased in the 14 patients who were diagnosed with achalasia compared with those in 14 healthy individuals. By contrast, Clayton *et al* (10) found no differences in the levels of TNF-α receptor, IL-6, IFN-γ, IL-12, IL-17, IL-22 and IL-23 in the serum of patients with achalasia compared with those with gastroesophageal reflux disease, in addition to those amongst the three subtypes of achalasia. However, Clayton *et al* (10) did not include a control group in their study. Due to these scarce and controversial findings, the present study detected and quantified the differences in the serum levels of a large panel of 27 biomarkers in 68 patients with achalasia, which were compared with those in the 39 healthy controls, with the aim of investigating the possibility of an inflammatory basis for the etiology of the achalasia. The serum protein levels of all markers in the patients with achalasia were found to be significantly lower compared with those in the control group, with the exception of IL-1 receptor antagonist, IL-4, IL-7, IL-9, eotaxin, IP-10 and PDGF, which did not show statistical significance. However, when the association between biomarker levels and clinical parameters of the patients with achalasia was analyzed, no differences were observed. At present, no explanation may be provided as to why higher inflammatory biomarker levels were found in the serum of the control cohort. Our working hypothesis was that the inflammatory profile observed in the inflammatory infiltrates in the tissue of patients with achalasia, which is considered to be the cause of reduction or loss of neurons, could also have been detected at systemic levels. The lack of elevations in the levels of inflammatory biomarkers tested in the circulation of patients, including cytokines, chemokines and growth factors, might indicate that achalasia is a disease that is caused by local inflammation. Specifically, the plasma biomarker levels may not accurately reflect the extent of tissue inflammation in one organ. Although the serum cytokine profiles of healthy subjects were previously investigated using magnetic bead-based multiplex immunoassays (26,27), the results from these previous studies are not completely comparable with those from the present study. Therefore, further studies with larger cohorts are required to corroborate these findings. Furthermore, variations in marker profiles identified amongst studies emphasizes the importance of considering differences in study designs and clinicopathological parameters of the participants, including inflammatory indices and inflammatory diseases that develop during data interpretation. In the present study, the control group included samples from healthy subjects collected from the Blood Bank of the Transfusion Service of the ‘Casa Sollievo della Sofferenza’ Hospital. As mentioned above, information on the serum cytokine profiles in achalasia is limited and therefore, the results of previous studies may not be entirely comparable with those from the present study. The apparent differences reported between the present and previous studies (9,10) may also be explained by the heterogeneity in the characteristics of the patients, the controls and the methodology used. In effect, although all authors applied immunoenzymatic assays for marker detection, the kits utilized were different. The present study utilized a large panel that integrated relevant cytokines and chemokines into a single assay, enabling the simultaneous interrogation of 27 biomarkers in a single well. This multiplex technology has been used to determine the presence and quantify the levels of underrepresented cytokines in an accurate, sensitive and reproducible manner (28)*.* By contrast, in the study by Clayton *et al* (10), each kit varied in sample amount and dilution, assay incubation times, volume of conjugates and substrates for all cytokines and receptors analyzed. They measured the levels of 7 markers, including IL-22 and IL-23, which were not tested in the present study and no control group was analyzed. The Human ELISA kit utilized by Wang *et al* (9) determined IL-17 and IL-22 levels, where it was only possible to compare IL-17 levels. Finally, since the present study was designed to characterize the systemic inflammation patterns in achalasia compared with those in controls, it did not analyze the immunohistochemical features of the inflammatory infiltrate within the myenteric plexus of the patients, which constitutes a limitation. Moreover, further validation using a larger sample size of sera from patients with achalasia is necessary to confirm the results of the present study.

In conclusion, the present results suggest that the inflammatory processes and the presence of an inflammatory infiltrate within the myenteric plexus of patients with achalasia observed previously appears to be a local event and is not reflected in the circulation. Therefore, further investigations should be undertaken to identify specific inflammatory biomarkers in serum samples that can contribute to the early diagnosis of the disease and to improve our understanding of the etiopathogenesis of achalasia.

## Supplementary Material

Frequency of observations with unquantified analytes concentration below and above the LOQ along with the corresponding replaced values.

## Figures and Tables

**Table I:** General characteristics of the patients and controls.

Characteristics	Patients	Controls
Sample size, n	68	39
Sex, n, male/female	35/33	20/19
Age, mean ± standard deviation	59±13	60±14
Dysphagia, n (%)	63 (92.6)	-
Esophageal regurgitation, n (%)	57 (83.8)	-
Chest pain, n (%)	11 (16.2)	-
Weight loss, n (%)		
<5	48 (70.6)	-
5-10	13 (19.1)	-
>10	7 (10.3)	-
Autoimmune condition, n (%)	18 (26.5)	-
Megaesophagus, n (%)	21(30)	-
Pneumatic dilatation, n (%)	18 (26.5)	-
Surgical myotomy, n (%)	35 (51.5)	-
Pneumatic dilatation and surgical myotomy, n (%)	15(22)	-

**Table II:** Concentration of proteins in the sera of patients with achalasia and controls.

Analyte	Controls, n=39^f^	Patients, n=68^f^	Raw P-value^a^	Bonferroni adjusted P-value^b^
IL-1β	2.49±0.97	1.07±0.52	<0.001^e^	<0.001^e^
IL-1Ra	5.16±0.83	4.70±0.76	0.005^d^	0.127
IL-2	2.77±0.51	1.91±0.49	<0.001^e^	<0.001^e^
IL-4	2.78±0.45	2.52±0.41	0.003^d^	0.082
IL-5	3.67±0.49	3.20±0.48	<0.001^e^	<0.001^e^
IL-6	2.72±0.71	1.58±1.09	<0.001^e^	<0.001^e^
IL-7	2.98±0.38	2.83±0.40	0.060	1.000
IL-8	5.52±1.39	2.53±0.91	<0.001^e^	<0.001^e^
IL-9	5.79±0.18	5.76±0.15	0.259	1.000
IL-10	2.31±0.51	1.64±1.02	<0.001^e^	0.006^d^
IL-12p70	1.37±0.93	0.38±0.64	<0.001^e^	<0.001^e^
IL-13	2.83±0.78	1.74±0.58	<0.001^e^	<0.001^e^
IL-15	5.09±0.58	4.11±0.46	<0.001^e^	<0.001^e^
IL-17	3.87±0.51	3.31±0.36	<0.001^e^	<0.001^e^
Eotaxin	5.71±0.48	5.68±0.47	0.769	1.000
FGF basic	3.73±0.31	3.52±0.18	<0.001^e^	<0.001^e^
G-CSF	7.30±0.68	6.17±0.43	<0.001^e^	<0.001^e^
GM-CSF	2.08±0.92	0.06±1.00	<0.001^e^	<0.001^e^
IFN-γ	2.75±1.15	0.67±1.14	<0.001^e^	<0.001^e^
IP-10	6.32±0.54	6.07±0.54	0.022^c^	0.602
MCP-1	4.58±0.62	3.94±0.57	<0.001^e^	<0.001^e^
MIP-1α	4.16±0.96	2.00±0.76	<0.001^e^	<0.001^e^
PDGF-BB	8.46±0.61	8.19±0.47	0.012^c^	0.323
MIP-1β	5.16±0.33	4.79±0.14	<0.001^e^	<0.001^e^
RANTES	9.95±0.50	9.41±0.59	<0.001^e^	<0.001^e^
TNF-α	4.10±0.37	3.58±0.27	<0.001^e^	<0.001^e^
VEGF	5.57±0.51	4.50±0.64	<0.001^e^	<0.001^e^

P-values from a two-sample t-test on log-transformed values for both

^a^unadjusted (raw) and

^b^adjusted for multiple comparisons using a Bonferroni correction.

^c^P<0.05,

^d^P<0.01,

^e^P<0.001.

^f^log(pg/ml), mean ± standard deviation. IL, interleukin; FGF basic, fibroblast growth factor basic; G-CSF, granulocyte colony-stimulating factor; GM-CSF, granulocyte-macrophage colony-stimulating factor; IFN, interferon; IP-10, interferon γ-induced protein 10; MCP, monocyte chemoattractant protein 10; MIP, macrophage inflammatory protein; PDGF-BB, platelet-derived growth factor BB; TNF, tumor necrosis factor; VEGF, vascular endothelial growth factor.

## Data Availability

The datasets used and/or analyzed during the present study are available from the corresponding author on reasonable request.
