# Supplementary material for: Circulating levels of cytokines, chemokines and growth factors in patients with achalasia
Source: Biomed Rep. 2021 Sep 10;15(5):92. doi: 10.3892/br.2021.1468 (PMC8461322; doi:10.3892/br.2021.1468)
Supplement: Frequency of observations with unquantified analytes concentration below and above the LOQ along with the corresponding replaced values. [file Supplementary_Data.pdf]

Table SI. Frequency of observations with unquantified analytes concentration below and above the LOQ along with the corresponding replaced values.

	LOQ<		LOQ>	
Analyte	n (%)	Replaced value	n (%)	Replaced value
IL-1β	1 (0.9)	0.711	0 (0.0)	-
IL-1Ra	0 (0.0)	-	0 (0.0)	-
IL-2	7 (6.2)	2.808	0 (0.0)	-
IL-4	0 (0.0)	-	0 (0.0)	-
IL-5	0 (0.0)	-	0 (0.0)	-
IL-6	18 (15.9)	1.449	0 (0.0)	-
IL-7	2 (1.8)	5.796	0 (0.0)	-
IL-8	0 (0.0)	-	0 (0.0)	-
IL-9	0 (0.0)	-	0 (0.0)	-
IL-10	9 (8.0)	0.846	0 (0.0)	-
IL-12p70	62 (54.9)	1.125	0 (0.0)	-
IL-13	5 (4.4)	1.917	0 (0.0)	-
IL-15	50 (44.3)	47.720	0 (0.0)	-
IL-17	0 (0.0)	-	0 (0.0)	-
Eotaxin	0 (0.0)	-	0 (0.0)	-
FGF basic	0 (0.0)	-	0 (0.0)	-
G-CSF	0 (0.0)	-	0 (0.0)	-
GM-CSF	49 (43.4.0)	0.585	0 (0.0)	-
IFN-γ	55 (48.7.0)	1.080	0 (0.0)	-
IP-10	0 (0.0)	-	0 (0.0)	-
MCP-1	0 (0.0)	-	0 (0.0)	-
MIP-1α	0 (0.0)	-	0 (0.0)	-
PDGF-BB	0 (0.0)	-	0 (00.)	-
MIP-1β	0 (0.0)	-	0 (0.0)	-
RANTES	0 (0.0)	-	42 (37.2%)	2,8206
TNF-α	0 (0.0)	-	0 (0.0)	-
VEGF	27 (23.9.0)	47.180	0 (0.0)	-

LOQ, limit of quantification; LOQ<, observations with unquantified concentrations lower than the minimum detectable. Replaced values were calculated as 10% lower than the minimum observed value; LOQ>, observations with unquantified concentrations greater than the maximum detectable. Replaced values were calculated as 10% higher the maximum observed value.
